# Gene expression meta-analysis in the prefrontal cortex: unraveling biological underpinnings of suicidal risk

**DOI:** 10.1186/s12888-026-08170-2

**Published:** 2026-05-20

**Authors:** Daniel F. Ramos-Rosales, Cristian A. Echeverria-Carrillo, Marcelo Barraza-Salas, Maribel Cervantes-Flores, Sergio M. Salas-Pacheco, Edna M. Méndez-Hernández, Francisco X. Castellanos-Juárez, Osmel La Llave-León, Ada A. Sandoval-Carrillo, José M. Salas-Pacheco

**Affiliations:** 1 Institute of Scientific Research, Juarez University of the State of Durango, Durango, Dgo. C.P. 34070 México; 2 Faculty of Chemical Sciences, Juarez University of the State of Durango, Durango, Dgo. C.P. 34070 México; 3 Faculty of Dentistry, Juarez University of the State of Durango, Durango, Dgo. C.P. 34070 México

**Keywords:** Suicide, Meta-analysis, Transcriptomic profile, DEG, Prefrontal cortex

## Abstract

**Background:**

Suicide is a complex public health challenge. Although psychosocial factors are important, molecular dysregulations in the prefrontal cortex (PFC) have been implicated in suicidal behavior.

**Methods:**

Six *post-mortem* PFC transcriptomic datasets from Gene Expression Omnibus (GEO) were analyzed to investigate the molecular basis of suicide, a major public health problem whose underlying mechanisms remain incompletely understood. After harmonizing microarray and sequencing data from 248 individuals younger than 60 years, both global and diagnosis-stratified meta-analyses were performed, focusing on major depressive disorder (MDD) and bipolar disorder (BPD).

**Results:**

In the overall comparison between suicide cases and controls, 10 nominally differentially expressed genes were identified, although none remained significant after multiple-testing correction. In the MDD-stratified analysis, four genes survived false discovery rate (FDR) correction (*HRH3*, *PDE2A*, *NET1*, and *RHBDF2*) and were associated with stress-related pathways, cyclic nucleotide signaling, and synaptic organization. No significant genes were identified in the bipolar disorder subgroup.

**Conclusion:**

These findings suggest that suicide-related transcriptomic alterations in the PFC are not uniform or clearly transdiagnostic, but may be partly shaped by the underlying psychiatric diagnosis, with a more clearly detectable signal in cases with MDD. This study provides a harmonized, bias-aware framework for integrating heterogeneous *post-mortem* transcriptomic datasets; however, the results should be interpreted as hypothesis-generating candidate signals rather than as definitive biomarkers, and require independent replication and functional validation.

**Supplementary Information:**

The online version contains supplementary material available at 10.1186/s12888-026-08170-2.

## Introduction

Suicide is a complex and multifaceted public health issue that imposes a significant burden on individuals, families, and societies worldwide. With over 700,000 deaths annually, it ranks as the 17th leading cause of mortality globally [1]. Beyond the tragic loss of life, suicide carries profound emotional, social, and economic consequences, highlighting the urgent need to unravel its underlying biological and psychological mechanisms. While environmental, psychological, and social factors play critical roles in suicidal behavior, increasing evidence suggests that genetic and molecular alterations also contribute significantly to suicide risk [2].

The PFC, a brain region essential for higher-order cognitive functions, has emerged as a key area of interest in suicide research because it regulates critical processes such as decision-making, emotional regulation, impulse control, and stress response [3, 4]. Dysregulation of these functions is frequently observed in individuals with psychiatric disorders, including major depressive disorder (MDD), bipolar disorder (BPD), and schizophrenia (SCZ), which are frequently associated with an increased risk of suicidal behavior. *Post-mortem* studies have revealed structural, functional, and molecular alterations in the PFC of individuals who died by suicide, including changes in genes linked to neuroplasticity, inflammation, the hypothalamic-pituitary-adrenal (HPA) axis, and neurotransmission [5, 6]. These findings suggest that the PFC may serve as a critical neural substrate for understanding the biological underpinnings of suicide.

Advances in genomic technologies and the availability of large-scale gene expression datasets in public repositories, such as GEO [7], have facilitated the investigation of molecular mechanisms underlying psychiatric disorders and suicidal behavior. Meta-analyses of these datasets enabled the identification of differentially expressed genes (DEGs) and dysregulated pathways implicated in suicide pathophysiology, providing valuable insights into potential molecular targets for further study [8, 9].

This study performed a cross-platform meta-analysis of post-mortem transcriptomic data from the PFC, integrating microarray and RNA-seq datasets harmonized at the *Ensembl* gene level. Suicide cases were defined as individuals classified in the GEO metadata as having died by suicide. Using a standardized bioinformatic framework with adjustment for available biological and technical covariates, we aimed to identify DEGs and associated functional patterns in both the global case–control comparison and diagnosis-stratified analyses, thereby providing a more confounder-aware molecular characterization of suicide in the PFC.

## Methods

### Data mining

A systematic search was conducted in September 2024 in the GEO DataSets repository (https://www.ncbi.nlm.nih.gov/geo/) to identify transcriptomic studies of suicide. The search combined Medical Subject Headings and keywords including “*prefrontal cortex*,” “*gene expression*,” “*microarray*,” “*RNA-seq*,” and “*suicide*.” Eligible studies were original experimental investigations that reported gene expression profiles in *post-mortem* human PFC tissue from individuals who died by suicide, compared with non-suicide controls. In this study, suicide cases were operationally defined as samples annotated in the GEO metadata as suicide deaths. Psychiatric status in the control group was not consistently available across the source GEO datasets; therefore, controls were retained according to the original study annotations as non-suicide controls. Datasets were included if they performed gene expression profiling using microarray or RNA-seq platforms, focused on the dlPFC or OFC, and provided key sample-level metadata including age, sex, and *post-mortem* interval (PMI). From the eligible datasets (microarray: GSE5388, GSE5389, GSE92538, and GSE208338; RNA-seq: GSE101521 and GSE248260), only samples from individuals younger than 60 years were included [10–14]. This age restriction was applied to minimize age-related transcriptomic heterogeneity in the PFC, as gene expression profiles undergo substantial and non-linear changes during ageing that can confound suicide-associated signals [15–17]. The number of excluded samples per dataset is provided in Supplementary Table S1. An overview of the analytical workflow is provided in Supplementary Figure S1, and the final proportion of samples included in the meta-analysis is presented in Table S1.

### Microarray data processing

Raw microarray data from CEL files were imported using the *affy* package (v1.88.0) for HG-U133A arrays and the *oligo* package (v1.74.0) for HuEx 1.0 ST arrays. For HG-U133A arrays, probe-level data were processed with the Robust Multi-array Average algorithm and quantile-normalized using *limma* (v3.66.0). Control probes matching the pattern “AFFX” were removed. Probes were mapped to Entrez identifiers and gene symbols, then converted to *Ensembl* gene (ENSG) identifiers using *org.Hs.eg.db* (v3.22.0). Expression data were merged with annotation and collapsed to the gene level by averaging probe intensities mapping to the same ENSG IDs, ignoring missing values. For HuEx 1.0 ST arrays, data were processed by Robust Multi-array Average at the “core” target level. Probes with expression values greater than 4.5 in at least 20% of samples were retained, followed by quantile normalization. Probe annotation was performed with *huex10sttranscriptcluster.db* (v8.8.0). Only probes with valid ENSG IDs were retained, and gene-level expression matrices were generated by averaging probe-level values.

### RNA-seq data processing

Raw RNA-seq data were downloaded from the Sequence Read Archive using the *SRA Toolkit* (v3.1.1) and converted to FASTQ files with fastq-dump using the --split-3 and --gzip options for paired-end reads. Sequence quality was assessed with FastQC. Adapter trimming and low-quality filtering were performed with *Trim Galore* (v0.6.4) using a minimum Phred score of 20 and a minimum read length of 30 nucleotides. Cleaned reads were pseudoaligned to the human reference transcriptome (GENCODE v47) with *Kallisto* (v0.51.1) using 100 bootstrap iterations and the expectation-maximization algorithm. Transcript-level abundance estimates were imported with *tximport* (v1.38.2) and summarized to gene-level counts using length-scaled TPM values. A tx2gene mapping was generated with *biomaRt* (v2.66.2) from *Ensembl* attributes after removal of missing, empty, and duplicate transcript-gene pairs. Transcript identifiers were matched independently of version suffixes, and version numbers were removed from ENSG IDs. Cross-platform integration was performed at the ENSG level after harmonization.

### Cross-platform transcriptomic and meta-analytic analysis

#### Data harmonization and preprocessing

Raw gene expression matrices from the four microarray and two RNA-seq studies were imported and harmonized at the ENSG IDs level after standardization of sample identifiers and exact alignment with metadata. Genes with missing or ENSG IDs were excluded. Duplicate entries were collapsed by summation for count data or averaging for normalized data. Samples with unmatched metadata or insufficient overlap were removed, and studies required at least six matched samples. Features with more than 50% missing values or variance ≤ 1e − 10 were excluded. RNA-seq matrices were retained as raw counts, while microarray matrices were analyzed as continuous expression values. Annotation from ENSG to gene symbols and names was obtained with *org.Hs.eg.db* through *AnnotationDbi* (v1.72.0). Unique mappings were retained, and missing annotations were replaced with ENSG identifiers.

#### Cell-type score estimation and principal component analysis (PCA) adjustment

Cell-type scores were estimated per sample from predefined marker gene sets [18–19] representing major neural and glial populations, requiring detection of at least three genes per cell type (Supplementary Table S2). Summary ratios were derived, and PCA was applied to the cell-type scores after low-variance filtering and row-median imputation, retaining up to two cell-type principal components per study.

#### Surrogate variable analysis (SVA)

Surrogate variable analysis was performed using the *sva* package (v3.58.0). Models included group, sex, age, *PMI*, pH, and available cell-type principal components. The number of surrogate variables was estimated with the “leek” method and constrained by sample size and model rank. For RNA-seq datasets, surrogate variable analysis was applied to log2-transformed counts per million, while microarray data were used directly. Genes with zero variance or insufficient observations were excluded before analysis, and non-finite values were imputed with row medians.

#### Study-specific differential expression analysis

Study-specific differential expression was analyzed independently for each study using *limma*. For RNA-seq data, counts were normalized with *edgeR* (v4.8.2) using calcNormFactors, filtered with filterByExpr, and transformed with voom using quality weights. Microarray data were modeled directly. Design matrices included group as the base variable and were extended stepwise with sex, age, PMI, pH, CellPCs, and surrogate variables only when at least six non-missing observations were available, sufficient variability existed, and full-rank model structure was preserved. Models were fitted with lmFit and empirical Bayes moderation. Genes with non-finite or zero standard errors were excluded. Study-specific results were harmonized by retaining common ENSG IDs across studies.

#### Cross-platform meta-analysis and quality control

Cross-study meta-analysis was performed using the *metafor* package (v4.8.0). Random-effects models were fitted with rma.uni using restricted maximum likelihood estimation, with fallback to the DerSimonian–Laird estimator when convergence failed. Genes were included only when valid effect sizes were available in at least four studies. Outputs included pooled effect sizes (meta_logFC), standard errors, z statistics, *p* values, confidence intervals, and heterogeneity metrics (τ², I², H², and Q-test *p* values). Multiple testing was controlled with the Benjamini–Hochberg FDR. Genomic inflation was evaluated with the lambda statistic and quantile-quantile plots.

#### Diagnosis-stratified cross-platform meta-analysis

A diagnosis-stratified cross-platform meta-analysis was performed separately for MDD and BPD after subsetting each study to controls and cases of the target diagnosis. Cell-type scores, CellPCs, and surrogate variables were recalculated within each diagnostic stratum. Study-specific differential expression analyses were repeated with adjustment for available covariates. Stratum-specific results were restricted to genes with valid effect sizes and standard errors, harmonized by intersection of ENSG IDs, and meta-analyzed with random-effects models requiring at least two independent studies per gene. Benjamini–Hochberg correction was applied. Nominal significance was defined as *p* < 0.05 with absolute log2 fold change (|logFC|) ≥ 0.2. This more stringent effect-size threshold was used to prioritize more pronounced diagnosis-specific signals within clinically more homogeneous subgroups. Genomic inflation was assessed with lambda statistics and quantile-quantile plots. SCZ was excluded because it was represented in only one dataset.

### Gene ontology enrichment analysis

Gene Ontology enrichment analysis was conducted with *topGO* (v2.62.0). For each dataset and ontology category (Biological Process “BP”, Cellular Component “CC”, and Molecular Function “MF”), a topGOdata object was created with annFUN.org and a node size of 5. Gene selection was defined by a binary indicator of DEGs. Enrichment was tested with Fisher’s exact test under the weight01 and elim algorithms. GO terms were ranked by the weight01 statistic, and the top 100 terms were extracted with GenTable. Terms with *p* ≤ 0.10 were retained for visualization, and the top 15 terms were selected according to significance and gene counts. −log10-transformed *p* values and gene ratios (Significant/Annotated) were calculated.

### Protein–protein interaction (PPI) network

A PPI network was constructed from the list of significant DEGs using the STRING database (v12.0). Interactions with a combined confidence score ≥ 0.4 were retained. The resulting network was analyzed to identify highly interconnected hub proteins.

### Compliance with PRISMA guidelines

This study adhered to the PRISMA 2020 guidelines for systematic reviews. Assessment of reporting biases (Item 21) was not performed using formal statistical methods due to inherent differences between RNA-seq and microarray platforms. No protocol was registered (Items 24a–c).

## Results

### Data characteristics and description of the study groups

Across the six datasets (Table 1), 248 samples were included, comprising 144 controls and 104 suicide cases. The median age was higher in controls than in suicide cases (*p* = 0.008), whereas PMI was longer in the suicide group (*p* = 0.001). Among suicide cases, the distribution of psychiatric diagnoses was 39.4% MDD, 36.6% SCZ, and 23.1% BPD (Table 2).

**Table 1:** Samples utilized in the meta-analysis

GEO Accession	Author	Brain Bank	Brain Region	Platform	Sample size use in meta-analysis
GSE5388	Ryan et al. [10]	SMRI-Brain Bank	dlPFC (BA 9)	Affymetrix Human Genome U133A Array	40: 9 BPD, 31 CNTRL
GSE5389	Ryan et al. [10]	SMRI-Brain Bank	OFC (BA 11)	Affymetrix Human Genome U133A Array	15: 5 BPD, 10 CNTRL
GSE92538	Hagenauer et al. [11]	Pritzker Consortium: UC-Irvine	dlPFC (BA 9/46)	Affymetrix Human Genome U133A Array	38: 5 BPD, 8 MDD, 25 CNTRL
GSE208338	Worf et al. [12]	Helmholtz Munich	dlPFC (BA 9)	Affymetrix Human Exon 1.0 ST Array	108: 5 BPD, 14 MDD, 38 SCZ, 56 CNTRL
GSE101521	Pantazatos et al. [13]	Columbia University	dlPFC (BA 9)	Illumina HiSeq 2500 - Paired	30: 9 MDD, 21 CNTRL
GSE248260	Sun et al. [14]	Icahn School of Medicine	OFC (BA 47)	Illumina HiSeq 2500 - Single	17: 10 MDD, 7 CNTRL

Abbreviations: dlPFC: Dorsolateral prefrontal cortex, OFC: Orbitofrontal cortex, BA: Brodmann area, BPD: Bipolar disorder, MDD: Major depressive disorder, CNTRL: Control, UC-Irvine: University of California–Irvine, SMRI: Stanley Medical Research Institute, SCZ: Schizophrenia

**Table 2:** Demographic data of the samples included in the meta-analysis

	Control	Suicides	*p*
	*n* = 144	*n* = 104	
**Sex**,** Male/ Female (n**,** %)**	117 (81.3) / 27 (18.8)	77 (74) / 27(26)	0.212 ^*x²*^
**Age (η**,** Q1-Q3)**	44, 34–52	36. 8, 28.5–48.92	**0.008** ^***u***^
**pH (µ ± SD)**	6.53 ± 0.299	6.52 ± 0.283	0.789 ^t^
**PMI (η**,** Q1-Q3)**	26, 16–38.75	35.5, 22–45.7	**0.001** ^***u***^
**Diagnosis (n**,** %)**			
**BPD**	N/A	24 (23.1)	
**MDD**	N/A	41 (39.4)	
**SCZ**	N/A	38 (36.5)	

BPD: bipolar disorder, MDD: major depressive disorder, N/A: not apply, PMI: *post-mortem* interval, SCZ, Q1-Q3: interquartile range, SCZ: schizophrenia, SD: standard deviation, ^*t*^: Student’s t-test, ^*u*^: Mann–Whitney U test, ^*x²*^ : Chi-squared test, η: median, µ: mean

### Study harmonization and quality assessment

After metadata standardization and quality filtering, 248 samples from six transcriptomic datasets were retained for cross-platform analysis: GSE208338 (*n* = 101), GSE5388 (*n* = 40), GSE5389 (*n* = 15), GSE92538 (*n* = 45), GSE101521 (*n* = 30), and GSE248260 (*n* = 17). Restriction to genes shared across all datasets yielded a final set of 9,863 ENSG features for meta-analysis.

Cell-type marker coverage was broad across all studies. Excitatory markers were detected in 11 of 12 genes in GSE208338 and in all 12 genes in the remaining datasets. Inhibitory, astrocyte, and pericyte markers showed complete coverage across studies, whereas microglial, oligodendrocyte, OPC, and endothelial markers were detected in 9/10 to 10/10, 7/8 to 8/8, 4/5 to 5/5, and 6/7 to 7/7 genes, respectively. PCA of the cell-type score matrix produced two cell-type principal components in every dataset.

Latent variable estimation identified no surrogate variables in GSE208338, GSE5388, GSE5389, GSE92538, or GSE248260, whereas one significant surrogate variable was detected in GSE101521. Global quality-control assessment showed that the meta-analytic test statistics were well calibrated, with a genomic inflation factor of 1.085 and deviation from the expected null distribution confined to the upper tail of the QQ plot (Supplementary Figure S2).

### Global transcriptomic meta-analysis

**Fig. 1:**
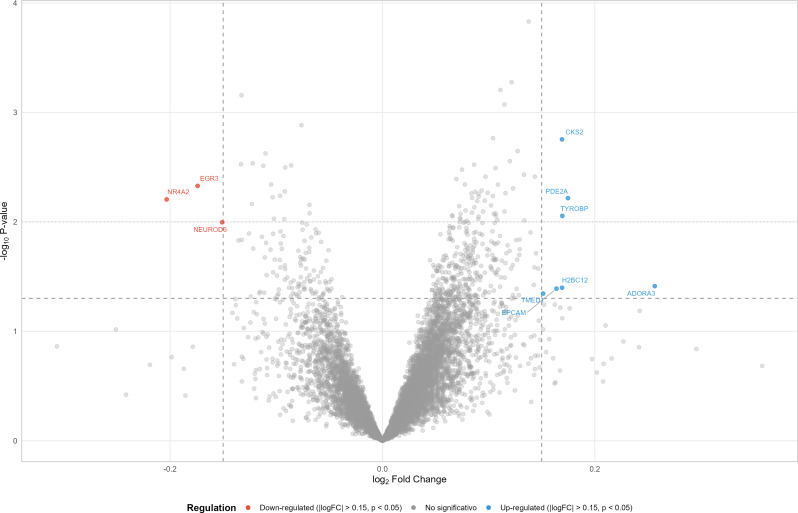
Volcano plot of nominally DEGs in the global cross-platform meta-analysis. Volcano plot showing gene expression changes in the global meta-analysis of 9,863 genes. Blue dots indicate upregulated genes and red dots indicate downregulated genes meeting the nominal thresholds of *p* < 0.05 and |logFC| > 0.15. Grey dots represent non-significant genes. Dashed lines indicate the significance and fold-change cutoffs

In the global cross-platform meta-analysis of 9,863 genes, 10 nominal DEGs were identified using the predefined thresholds of *p* < 0.05 and |logFC| > 0.15, although none remained significant after multiple-testing correction (all adjusted *p* = 0.9376). These included seven upregulated genes (*CKS2*, *PDE2A*, *TYROBP*, *H2BC12*, *TMED1*, *EPCAM*, and *ADORA3*) and three downregulated genes (*EGR3*, *NR4A2*, and *NEUROD6*). *ADORA3* showed the largest positive effect size (logFC = 0.256), whereas *NR4A2* showed the largest negative effect size (logFC = -0.203) (Table 3; Fig. 1). Heterogeneity across the 10 nominal genes ranged from I² = 0.0% for *NR4A2* to 86.2% for *ADORA3* (Supplementary Table S3). No diagnosis-related differences were detected when the global nominal genes were compared across MDD, BPD, and SCZ case samples using study-adjusted omnibus models (Supplementary Figure S3).

**Table 3:** Nominally DEGs identified in the global cross-platform meta-analysis

Symbol	Gene name	Log FC	*p*	Adj. *p*	Regulation
*CKS2*	CDC28 protein kinase regulatory subunit 2	0.169	0.001	0.937	Upregulated
*EGR3*	early growth response 3	-0.174	0.004	0.937	Downregulated
*PDE2A*	phosphodiesterase 2 A	0.174	0.006	0.937	Upregulated
*NR4A2*	nuclear receptor subfamily 4 group A member 2	-0.203	0.006	0.937	Downregulated
*TYROBP*	transmembrane immune signaling adaptor TYROBP	0.169	0.008	0.937	Upregulated
*NEUROD6*	neuronal differentiation 6	-0.150	0.010	0.937	Downregulated
*ADORA3*	adenosine A3 receptor	0.256	0.038	0.937	Upregulated
*H2BC12*	H2B clustered histone 12	0.169	0.040	0.937	Upregulated
*EPCAM*	epithelial cell adhesion molecule	0.163	0.040	0.937	Upregulated
*TMED1*	transmembrane p24 trafficking protein 1	0.151	0.045	0.937	Upregulated

### Diagnosis-stratified transcriptomic profiles

In the diagnosis-stratified analyses, the BPD versus control contrast evaluated 10,510 genes and identified 79 nominally DEGs with |logFC| > 0.2 and *p* < 0.05, including 8 upregulated and 71 downregulated genes; none remained significant after FDR correction. The most significant nominal BPD DEGs included *ARPP19*, *HMGCS1*, *ECHDC1*, *MBNL2*, *EDIL3*, *UGT8*, and *WFS1* (Supplementary Table S4; Supplementary Figure S4).

In the MDD versus control contrast, 9,863 genes were evaluated and 74 nominal DEGs were identified at the same threshold, including 59 upregulated and 15 downregulated genes (Supplementary Table S5; Supplementary Figure S5). Among the nominal MDD signals, *EFNB1*, *ARRB2*, *AHDC1*, *SECTM1*, *NNAT*, *G0S2*, *INTS13*, *PAMR1*, and *CTNNA3* showed low *p* values. Four genes remained significant after FDR correction: *HRH3*, *PDE2A*, *NET1*, and *RHBDF2* (Supplementary Table S6).

Overlap with the global meta-analysis was limited. *PDE2A* and *TYROBP* were retained in the nominal MDD list, whereas *PDE2A* was the only gene that overlapped between the FDR-significant MDD results and the nominal global DEG set. *TMED1* was the only overlapping gene in the nominal BPD list (Supplementary Figure S6). All genes retained in the diagnosis-stratified meta-analyses were supported by four contributing studies. Heterogeneity among the FDR-significant genes identified in the MDD-stratified analysis was minimal, with I² values ranging from 0% to 7.9% and non-significant Q-test *p* values in all cases (Supplementary Table S6). Quantile-quantile plots for the BPD- and MDD-stratified meta-analyses are shown in (Supplementary Figures S7).

### Gene ontology enrichment analysis

**Fig. 2:**
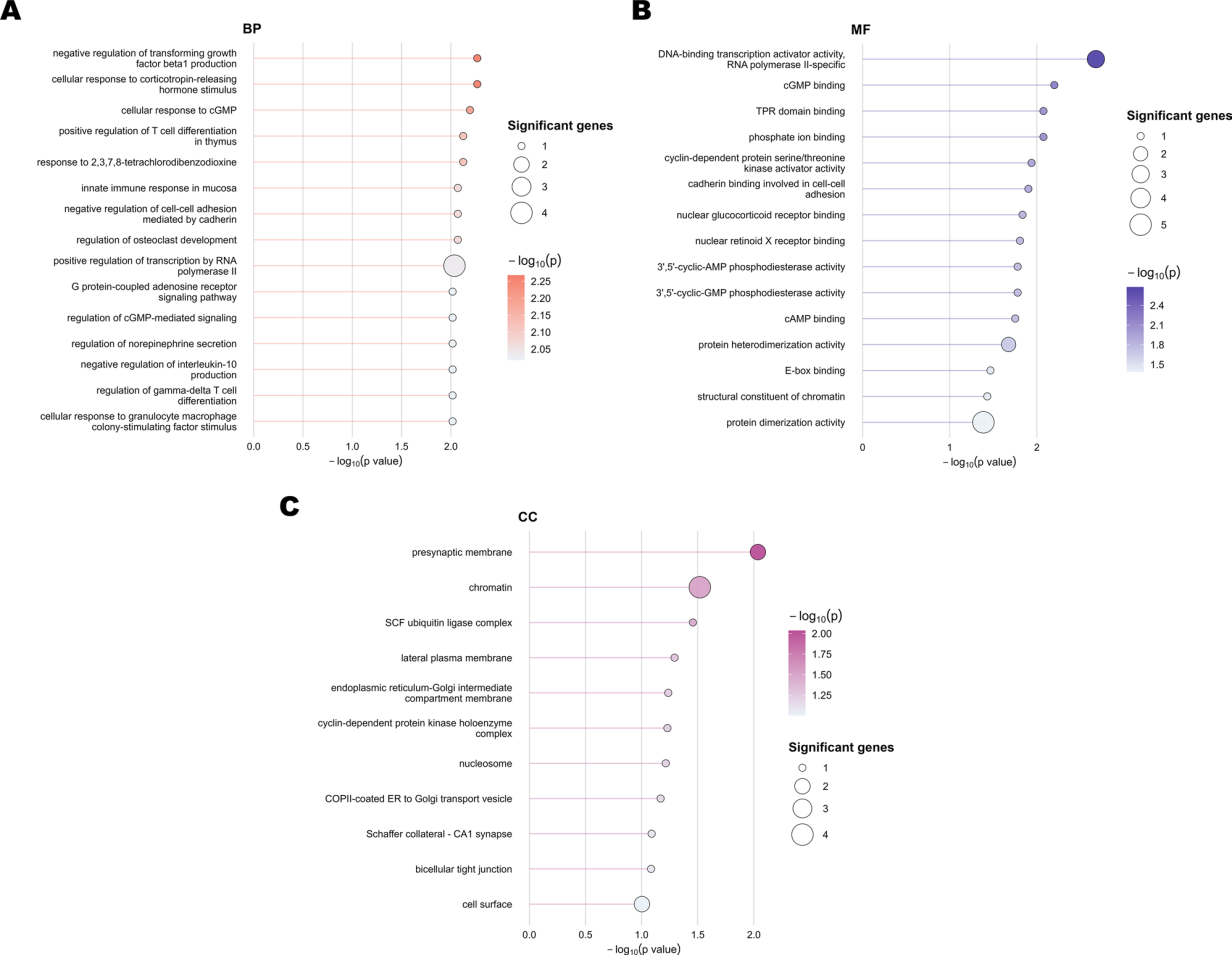
Gene Ontology enrichment of nominally differentially expressed genes in the global meta-analysis. Gene Ontology enrichment results for the nominally DEGs identified in the global meta-analysis, shown for (**A**) BP, (**B**) MF, and (**C**) CC. Bubble size represents the number of genes associated with each term, and color intensity indicates enrichment significance

Among BP terms, the leading categories included *negative regulation of transforming growth factor beta1 production*, *cellular response to corticotropin-releasing hormone stimulus*, and *cellular response to cGMP* (Fig. 2-A). Among MF terms, the highest-ranked categories included *DNA-binding transcription activator activity*, *RNA polymerase II-specific*,* cGMP binding*, and *TPR domain binding* (Fig. 2-B). In the CC ontology, *presynaptic membrane* and *chromatin* were among the top-ranked categories (Fig. 2-C).

Diagnosis-stratified enrichment analyses are shown in the Supplementary Figures S8 and S9; in BPD, the top-ranked terms included *neural tube closure*, *cAMP-dependent protein kinase complex*, and *protein kinase A catalytic subunit binding*, whereas in MDD they included *host-mediated suppression of symbiont invasion*, *blood microparticle*, and *epidermal growth factor receptor binding*.

### PPI networks

**Fig. 3:**
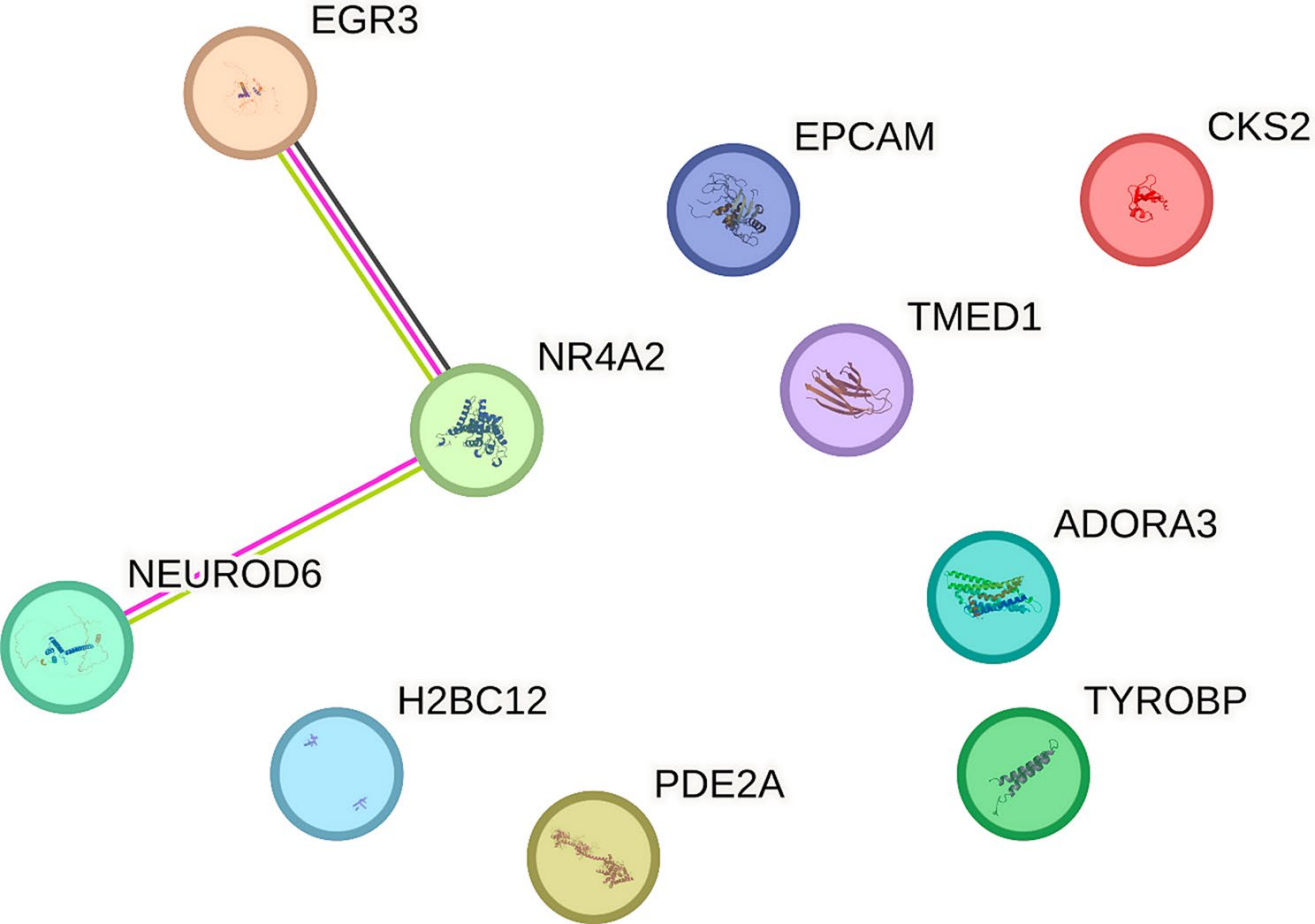
Protein–protein interaction network of nominally differentially expressed genes in the global meta-analysis. Network constructed from the nominally DEGs using STRING (combined score ≥ 0.4). Nodes represent genes and edges represent predicted or known protein–protein interactions

PPI analysis of the global nominal DEG set showed limited connectivity. At the retained STRING threshold (combined score ≥ 0.4), only two interactions were observed, forming a single connected component comprising NR4A2, EGR3, and NEUROD6. NR4A2 was the only node with more than one connection, linking separately to EGR3 and NEUROD6 (Fig. 3).

The MDD-stratified network showed a larger connected structure centered on CSF1R, CD74, HSPA1B, TYROBP, and SPP1, whereas the BPD-stratified network included a principal connected component containing FOS, PIK3CA, NRAS, PRKACB, and HMGCS1, together with smaller subnetworks and isolated nodes (Supplementary Figures S10 and S11).

## Discussion

Our cross-platform meta-analysis integrated six *post-mortem* PFC transcriptomic datasets harmonized at the *Ensembl* gene level and adjusted for available biological and technical covariates, including sex, age, PMI, brain pH, cell-type principal components, and surrogate variables where applicable. The global analysis revealed only a modest transcriptomic signal associated with suicide, with ten nominally significant DEGs identified at the predefined threshold (*p* < 0.05 and |logFC| > 0.15), although none remained significant after FDR correction (*CKS2*, *PDE2A*, *TYROBP*, *H2BC12*, *TMED1*, *EPCAM*, *ADORA3*, *EGR3*, *NR4A2*, and *NEUROD6*). In contrast, diagnosis-stratified analyses revealed more distinct transcriptional patterns, particularly in the MDD stratum, where four genes (*HRH3*, *PDE2A*, *NET1*, and *RHBDF2*) remained significant after multiple-testing correction. Collectively, these findings suggest that transcriptomic alterations associated with suicide in the PFC are heterogeneous and partly conditioned by the underlying psychiatric diagnosis rather than reflecting a uniform signal across affective and psychotic disorders [9, 20]. Given that the global analysis identified only nominally significant genes and the diagnosis-stratified findings have not yet been validated in an independent cohort, these results should be interpreted as candidate molecular signals.

The lack of FDR-significant findings in the global analysis should be interpreted as informative rather than simply negative. Previous transcriptomic studies of suicide in the PFC reported similarly limited or variable signals when different diagnostic groups are pooled together. Our results align with this pattern. For example, a large RNA-seq study found that individuals who died by violent suicide showed transcriptomic profiles in the dlPFC that differed more from other psychiatric patients than from healthy controls, highlighting the role of both diagnosis and features of the suicidal act [21]. A recent meta-analysis reached a comparable conclusion, noting that shared gene expression signals tend to be diluted in clinically heterogeneous samples [9].

The emergence of clearer signals after diagnostic stratification, with FDR-significant genes restricted to the MDD comparison, supports the view that suicide-associated transcriptomic alterations are shaped by the underlying psychiatric context. Within this stratum, *PDE2A* was one of four genes that survived FDR correction and the only one that also appeared among the nominal DEGs in the global meta-analysis, making it one of the most consistent findings in the study. PDE2A regulates cyclic nucleotide turnover in the central nervous system, and preclinical studies have shown that its inhibition enhances cAMP/cGMP signaling and markers of synaptic plasticity, supporting its biological relevance to mood-related phenotypes [22, 23]. At the same time, interpretation should remain cautious, as previous *post-mortem* work indicates that *PDE2A* expression varies according to brain region, psychiatric diagnosis, and sex rather than following a uniform disease pattern [24]. This context dependence is consistent with the findings of Cabrera et al., in which *PDE2A* did not emerge as an isolated signal but as part of a downregulated gene set linked to cation transport, neuronal components, signaling, and synaptic function in dlPFC from suicides with comorbid substance use disorder compared with suicides without substance use disorder, and was also represented in broader cell differentiation categories [25].

Among the nominally upregulated genes in our global meta-analysis, *TYROBP* was notable because it also emerged in the nominal MDD-specific signal and held a central position in the MDD-stratified PPI network, providing internal consistency across analyses. TYROBP (formerly DAP12) encodes a microglia-enriched adaptor protein involved in receptor-mediated signaling pathways that regulate microglial activation, phagocytosis, and innate immune responses in the central nervous system [26, 27]. In neurodegenerative models, particularly Alzheimer’s disease, the TREM2–TYROBP/DAP12 axis has been strongly linked to disease-associated microglial states and immune-inflammatory processes, lending biological plausibility to its detection in *post-mortem* brain tissue [28]. In our study, TYROBP may represent a biologically plausible signal of altered microglial or innate immune activity in the PFC. This interpretation is consistent with prior *post-mortem* studies that have identified *TYROBP* among candidate genes in suicide-related analyses within SCZ, although the available evidence remains insufficient to support a robust, diagnosis-independent association with suicide [29].

Among the other nominally upregulated DEGs (*CKS2*, *H2BC12*, *TMED1*, *EPCAM*, and *ADORA3*), EPCAM warrants brief mention because it was reported as the strongest putamen eQTL associated with rs184204326, a variant in *FBXO11* linked to suicidal behaviors and alcohol use disorder severity in an American Indian population [30]. However, at present, these genes remain only weakly supported in the context of suicide.

On the other hand, although *EGR3* and *NR4A2* have not been consistently established as suicide-related transcriptomic markers in *post-mortem* brain tissue, their downregulation in the global meta-analysis is biologically noteworthy. Together with *NEUROD6*, these genes formed the only connected component in the global PPI network, suggesting that this pattern reflects a coherent neuronal module rather than isolated signals. EGR3 is an activity-dependent transcription factor involved in stress-responsive gene regulation and synaptic plasticity, whereas NR4A2 is a nuclear receptor with well-established roles in neuronal regulation, particularly in dopaminergic function, and its genetic variation has been linked to depressive symptom dimensions and antidepressant response [31–34]. NEUROD6 has also been reported to be reduced in dlPFC pyramidal neurons in SCZ. supports the interpretation that this signal may reflect alterations in neuronal plasticity, activity-dependent transcriptional regulation, or stress-sensitive cortical circuitry in suicide. However, these genes cannot yet be considered established or suicide-specific transcriptomic markers.

Although the global neuronal signal was modest and exploratory, diagnostic stratification revealed a clearer pattern, particularly in the MDD subgroup, where *HRH3* was one of the four genes that remained significant after multiple-testing correction. *HRH3* is biologically plausible in this setting, although it cannot yet be considered an established transcriptomic marker of suicide. The histamine H3 receptor functions as a presynaptic auto- and heteroreceptor that regulates histamine release and modulates other neurotransmitter systems relevant to affective regulation, including serotonin, norepinephrine, and acetylcholine. Consistent with this role, preclinical studies have shown that chronic H3 receptor antagonism exerts antidepressant- and anxiolytic-like effects and restores BDNF expression in the PFC and hippocampus under chronic stress conditions [35]. At the same time, the available human transcriptomic evidence remains limited. In a recent candidate-gene meta-analysis of 12 *post-mortem* bulk transcriptomic datasets, Martins et al. reported broader histaminergic involvement in suicide but did not find significant differential expression for *HRH3* itself (effect size = 0.209, *p* = 0.121), suggesting that any contribution of *HRH3* may be context-dependent rather than consistently detectable at the bulk mRNA level; however, these findings are currently available only as a preprint [36]. Against this background, our finding that HRH3 remained significant after FDR correction in the MDD-stratified analysis should be interpreted as a diagnosis-specific signal that is biologically credible, but still requires independent replication before stronger conclusions can be drawn. By contrast, *NET1* and *RHBDF2* should presently be considered promising but exploratory signals that require independent replication before stronger functional claims are made.

The global GO profile was modest but biologically coherent. Across ontologies, enriched terms converged on three broad themes: stress-related neuroendocrine processes, including CRH response and glucocorticoid receptor binding; cyclic nucleotide/GPCR signaling, including cGMP-related signaling, cAMP binding, phosphodiesterase activity, and adenosine receptor pathways; and synaptic-transcriptional organization, including presynaptic membrane, chromatin, nucleosome, and RNA polymerase II-specific transcription factor activity. This pattern is consistent with postmortem evidence of altered CRH- and GABA_𝐴_-related transcripts in PFC [37], increased CRF expression in suicide PFC [38], glucocorticoid receptor-related epigenetic changes [39], abnormal G-protein signaling [40], and broader glutamatergic/GABAergic transcriptomic alterations in suicide and depression [41, 42].

In the BPD-stratified analysis, the nominal DEG profile was predominantly downregulated, aligning with GO/PPI results on synaptic signaling dysregulation, PKA/cAMP abnormalities, and myelin biology [10, 43–47]. Signals involving *PRKACB*, *PRKAR1A*, *SCRIB*, *UGT8*, and *WFS1* tentatively suggest suicide-relevant biology, though evidence is limited [46, 48, 49]. Since no DEG survived correction, findings remain exploratory.

By contrast, the MDD-stratified nominal profile was more coherent, extending the FDR-significant signals for HRH3 and PDE2A and a broader immune/stress-related network including *CSF1R*, *CD74*, *TYROBP*, *SPP1*, and *HSPA1B*. Together with the GO results, this pattern is consistent with cyclic nucleotide signaling abnormalities and glial-inflammatory changes previously reported in the PFC of individuals who died by suicide and depressed suicide subjects [46, 50–53], although HRH3-specific postmortem evidence in suicide remains limited.

Our findings are best interpreted in relation to the methodological diversity of prior suicide brain meta-analyses rather than through strict gene-by-gene concordance alone. Cabrera-Mendoza et al.. approached the question as a transcription factor network meta-study based on publicly available PFC datasets from suicide and non-suicide cases across MDD, BPD, SCZ, and healthy controls, emphasizing regulatory architecture rather than pooled differential-expression estimates [8]. Piras et al.. subsequently performed a region-stratified meta-analysis of publicly available *post-mortem* brain expression datasets, conducting four separate meta-analyses across OFC, dlPFC/PFC, dlPFC alone, and a combined frontal-cortex framework [9]. More recently, Sokolov et al. expanded the field toward a broader multiverse design by integrating multiple cohorts across brain regions and transcriptomic modalities while explicitly comparing unadjusted, covariate-adjusted, SVA, and cell-type-informed analytical strategies [54]. Against this background, the present study differs methodologically by restricting the analysis to PFC samples from individuals younger than 60 years, applying cross-platform integration at the ENSG level, and implementing harmonized study-wise differential-expression modeling with adjustment for age, sex, brain pH, PMI, cell-type principal components, and surrogate variables where applicable. In addition, the same analytical pipeline was repeated after restricting the case group to suicide cases with MDD or suicide cases with BPD, while retaining the full control group in each comparison. In this sense, the principal contribution of the present work lies not merely in providing an additional list of DEGs, but in offering a more standardized and confounder-aware analytical framework for interpreting heterogeneous *post-mortem* suicide transcriptomic data.

Several limitations should be considered when interpreting these findings. Despite a deliberately stringent analytical pipeline, the effective sample size remained modest (248 samples across six datasets), which limited statistical power to detect small but reproducible effects and is consistent with the largely nominal nature of the global meta-analysis. Between-study heterogeneity was substantial, reflecting differences in cohort composition, platform characteristics, and the uneven availability of clinical and technical metadata (a common challenge in prior suicide brain transcriptomic meta-analyses). The restriction to PFC samples from individuals younger than 60 years enhanced cohort homogeneity but necessarily limits generalizability to other brain regions and older age groups. Although we adjusted for age, sex, brain pH, PMI, cell-type principal components, and surrogate variables, residual confounding from medication exposure, substance use, agonal factors, trauma history, and other unmeasured clinical variables cannot be excluded. In addition, RNA integrity number (RIN), an important indicator of RNA quality in *post-mortem* transcriptomic studies, could not be incorporated into the harmonized models because it was not available in a sufficiently complete and standardized form across all included datasets. Cross-platform harmonization at the ENSG level improved comparability at the cost of reduced transcriptomic coverage and potential loss of isoform-specific signals. Likewise, cell-type adjustment relied on inferred bulk-tissue scores rather than formal deconvolution or single-cell data, constraining cell-specific interpretation. Importantly, the number of robustly supported genes was limited: the global meta-analysis yielded only nominally significant genes, none of which survived FDR correction, whereas FDR-significant findings were restricted to four genes in the MDD-stratified analysis. These diagnosis-stratified signals were not tested in an independent external cohort and therefore should be interpreted as prioritized candidate genes rather than validated suicide- or disease-specific biomarkers. In addition, no experimental validation, such as qPCR, immunohistochemistry, or in vitro assays. Finally, the inability to perform a SCZ-stratified analysis due to only one eligible dataset and the absence of independent experimental validation mean that the present results should be viewed primarily as statistically informed, hypothesis-generating observations that require replication in larger, better-annotated, independent, and functionally validated cohorts.

## Conclusion

Taken together, our findings suggest that suicide-related transcriptomic variation in the prefrontal cortex is modest at the global level and appears to be shaped, at least in part, by underlying psychiatric diagnosis. Although clinically heterogeneous pooling yielded limited shared signals, diagnostic stratification, particularly in suicide-MDD, revealed a more coherent profile that included *HRH3*, *PDE2A*, *NET1*, and *RHBDF2*. By integrating microarray and RNA-seq data at the ENSG level and adjusted for available biological and technical covariates, cell-type, and latent technical factors, this study provides a more standardized framework for analyzing heterogeneous *post-mortem* datasets. These results do not establish definitive biomarkers of suicide, but they support a more context-sensitive model of suicide neurobiology. Future studies should evaluate these signals in larger, better-annotated, regionally diverse, and functionally validated cohorts using transcript-level and cell-resolved approaches.

## Supplementary Information

Below is the link to the electronic supplementary material.

Supplementary Material 1: Analytical workflow of the cross-platform transcriptomic meta-analysis.

Supplementary Material 2: Quantile–quantile plot of the global meta-analysis. QQ plot comparing observed and expected –log10(p) values from the global meta-analysis. The dashed line indicates the expected null distribution, and lambda denotes the genomic inflation factor.

Supplementary Material 3: Diagnosis-specific expression distribution of global nominal genes. Boxplots showing within-study z-scored expression levels of the nominally differentially expressed genes from the global meta-analysis across major depressive disorder (MDD), bipolar disorder (BPD), and schizophrenia (SCZ) case samples. Points represent individual samples, and the reported p and FDR values correspond to study-adjusted omnibus comparisons across diagnostic groups.

Supplementary Material 4: Volcano plot of the BPD-stratified meta-analysis. Volcano plot showing gene expression changes in the BPD versus control meta-analysis. Blue dots indicate upregulated genes and red dots indicate downregulated genes meeting the nominal thresholds of p < 0.05 and |logFC| > 0.2. Grey dots represent non-significant genes. Dashed lines indicate the significance and fold-change cutoffs.

Supplementary Material 5: Volcano plot of the MDD-stratified meta-analysis. Volcano plot showing gene expression changes in the MDD versus control meta-analysis. Dark blue dots indicate genes that remained significant after FDR correction, whereas light blue and red dots indicate nominally significant upregulated and downregulated genes meeting the thresholds of p < 0.05 and |logFC| > 0.2. Grey dots represent non-significant genes. Dashed lines indicate the significance and fold-change cutoffs.

Supplementary Material 6: Overlap of nominal and FDR-significant differentially expressed genes across global and diagnosis-stratified analyses. Venn diagram showing the overlap among nominally differentially expressed genes identified in the global meta-analysis, the BPD-stratified analysis, and the MDD-stratified analysis, together with the FDR-significant genes from the MDD-stratified analysis.

Supplementary Material 7: Quantile–quantile plots of the diagnosis-stratified meta-analyses. QQ plots comparing observed and expected –log10(p) values for (A) the BPD versus control meta-analysis and (B) the MDD versus control meta-analysis. Dashed lines indicate the expected null distribution, and λGC denotes the genomic inflation factor.

Supplementary Material 8: Gene Ontology enrichment analysis of nominally differentially expressed genes in the BPD-stratified meta-analysis. Gene Ontology enrichment results for the nominally differentially expressed genes identified in the BPD-stratified meta-analysis, shown for (A) BP, (B) MF, and (C) CC. Bubble size represents the number of genes associated with each term, and color intensity indicates enrichment significance.

Supplementary Material 9: Supplementary Figure S8. Gene Ontology enrichment analysis of nominally differentially expressed genes in the MDD-stratified meta-analysis. Gene Ontology enrichment results for the nominally differentially expressed genes identified in the MDD-stratified meta-analysis, shown for (A) BP, (B) MF, and (C) CC. Bubble size represents the number of genes associated with each term, and color intensity indicates enrichment significance.

Supplementary Material 10: PPI network of nominally differentially expressed genes in the MDD-stratified meta-analysis. Network generated with STRING using the nominally differentially expressed genes identified in the MDD versus control meta-analysis and a combined interaction score threshold of ≥ 0.4. Nodes represent genes and edges represent predicted or known protein–protein interactions.

Supplementary Material 11: PPI network of nominally differentially expressed genes in the BPD-stratified meta-analysis. Network generated with STRING using the nominally differentially expressed genes identified in the BPD versus control meta-analysis and a combined interaction score threshold of ≥ 0.4. Nodes represent genes and edges represent predicted or known protein–protein interactions.

Supplementary Material 12

## Data Availability

Data are available upon reasonable request to the corresponding author.

## Abbreviations

BPD: Bipolar disorder
CNTRL: Control
DEG: Differentially expressed gene
dlPFC: Dorsolateral prefrontal cortex
ENSG: Ensembl gene identifier
FDR: False discovery rate
GEO: Gene Expression Omnibus
GO: Gene Ontology
MDD: Major depressive disorder
OFC: Orbitofrontal cortex
PFC: Prefrontal cortex
PMI: Post-mortem interval
PPI: Protein–protein interaction
PRISMA: Preferred Reporting Items for Systematic Reviews and Meta-Analyses
RNA-seq: RNA sequencing
SCZ: Schizophrenia
STRING: Search Tool for the Retrieval of Interacting Genes/Proteins
SVA: Surrogate variable analysis
