# Supplementary figures and images for: Gene expression meta-analysis in the prefrontal cortex: unraveling biological underpinnings of suicidal risk
Source: BMC Psychiatry. 2026 May 20;26:545. doi: 10.1186/s12888-026-08170-2 (PMC13390219; doi:10.1186/s12888-026-08170-2)

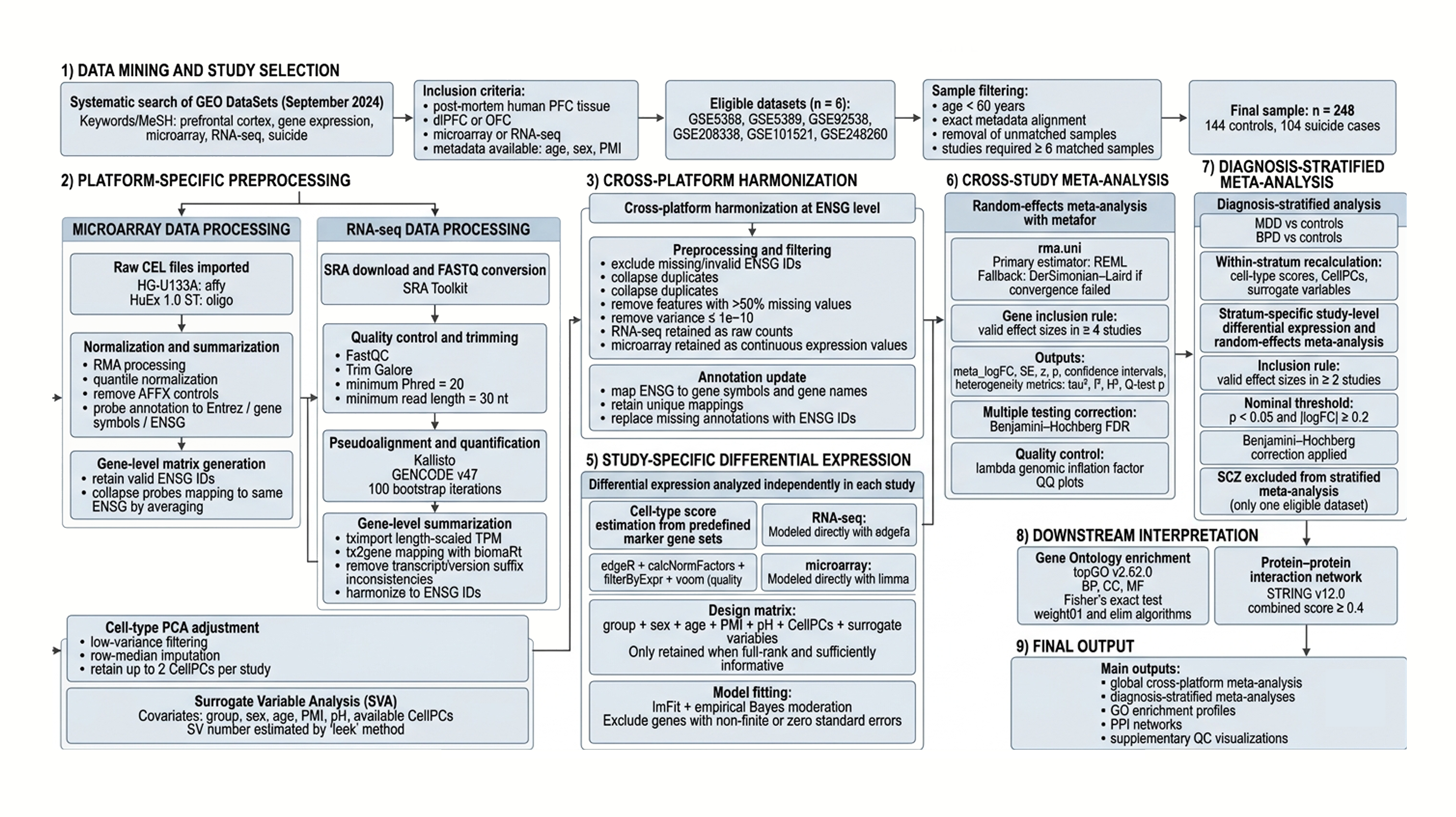

Supplement: Supplementary file 1 — Supplementary Material 1: Analytical workflow of the cross-platform transcriptomic meta-analysis. [file 12888_2026_8170_MOESM1_ESM.png]

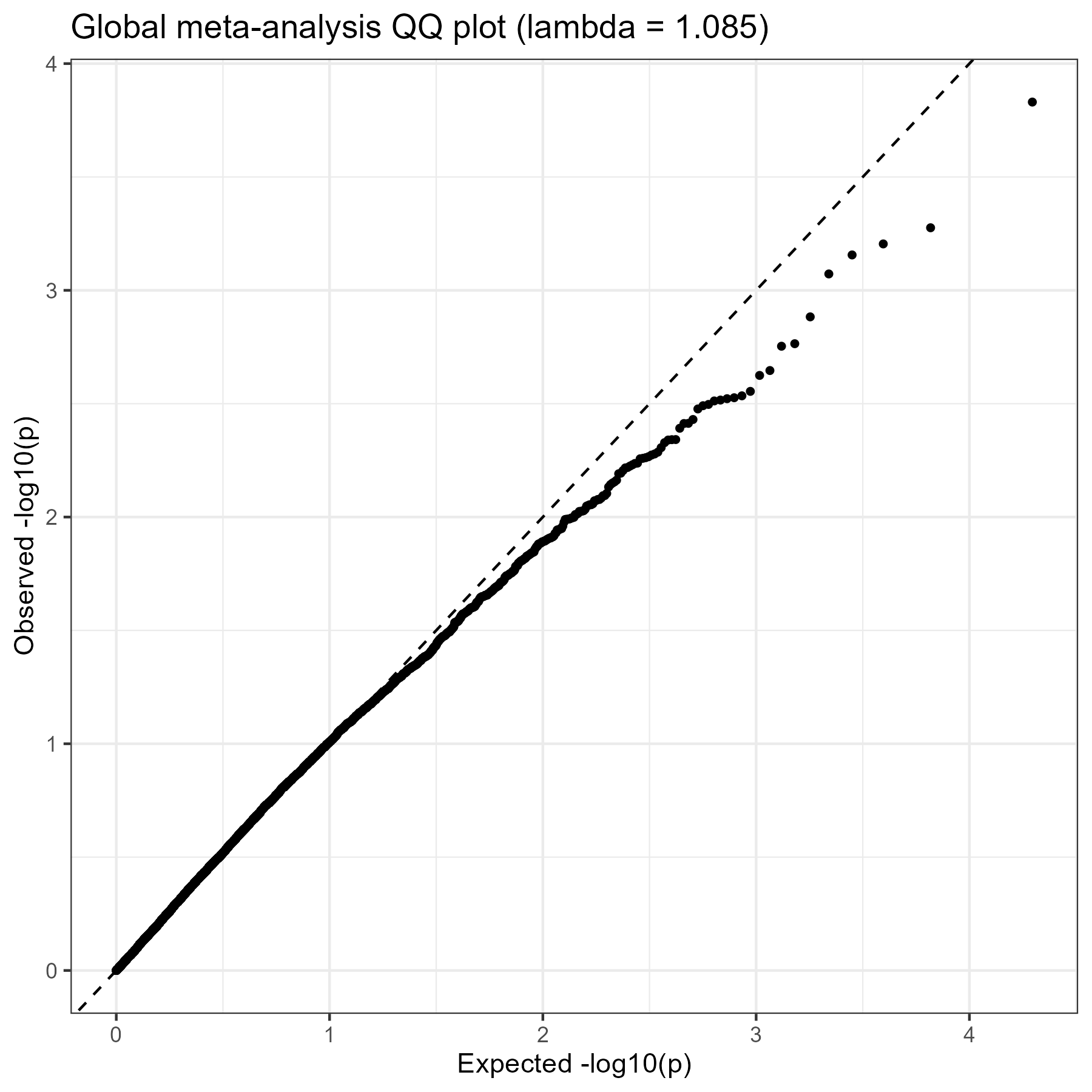

Supplement: Supplementary file 2 — Supplementary Material 2: Quantile–quantile plot of the global meta-analysis. QQ plot comparing observed and expected –log10(p) values from the global meta-analysis. The dashed line indicates the expected null distribution, and lambda denotes the genomic inflation factor. [file 12888_2026_8170_MOESM2_ESM.png]

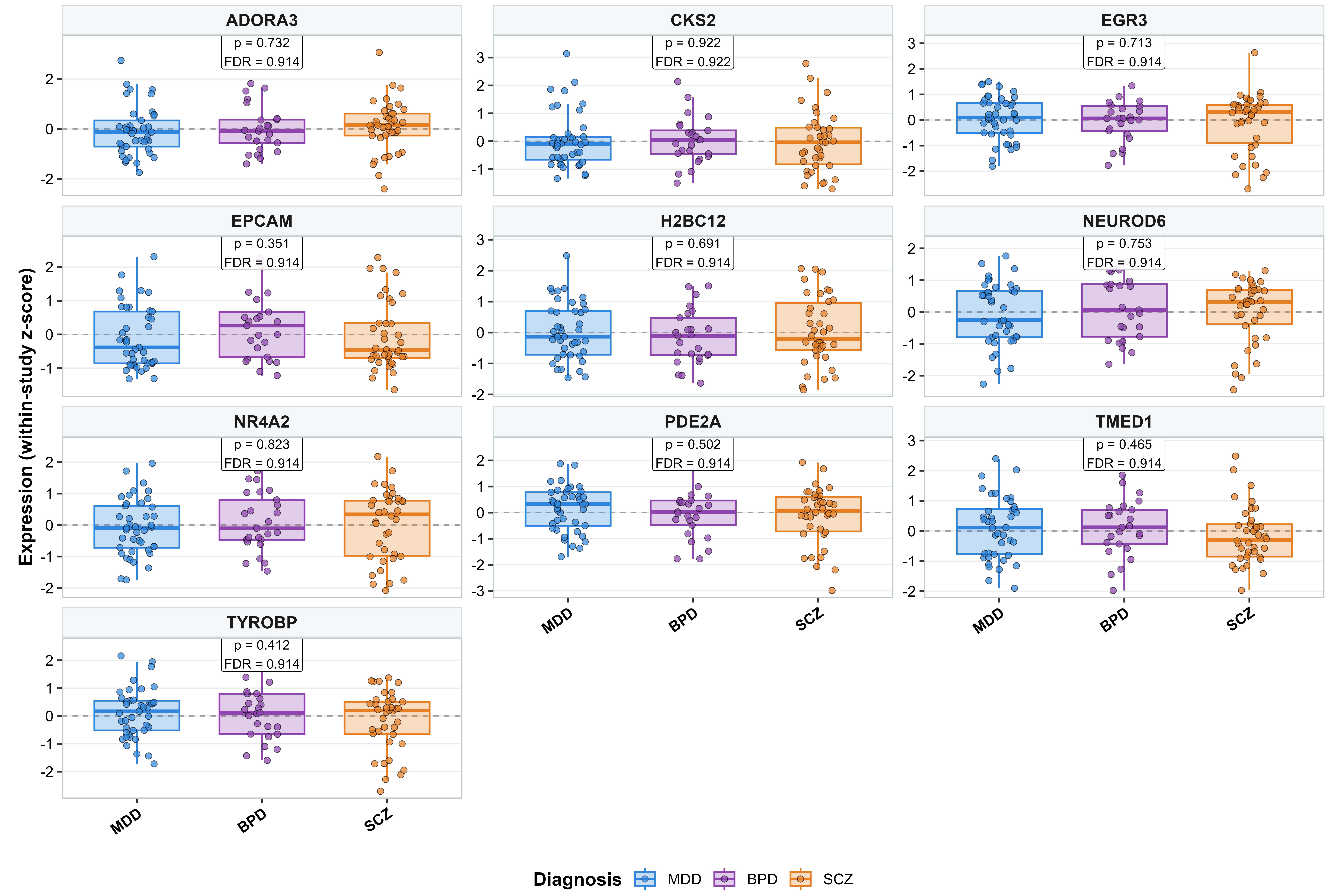

Supplement: Supplementary file 3 — Supplementary Material 3: Diagnosis-specific expression distribution of global nominal genes. Boxplots showing within-study z-scored expression levels of the nominally differentially expressed genes from the global meta-analysis across major depressive disorder (MDD), bipolar disorder (BPD), and schizophrenia (SCZ) case samples. Points represent individual samples, and the reported p and FDR values correspond to study-adjusted omnibus comparisons across diagnostic groups. [file 12888_2026_8170_MOESM3_ESM.png]

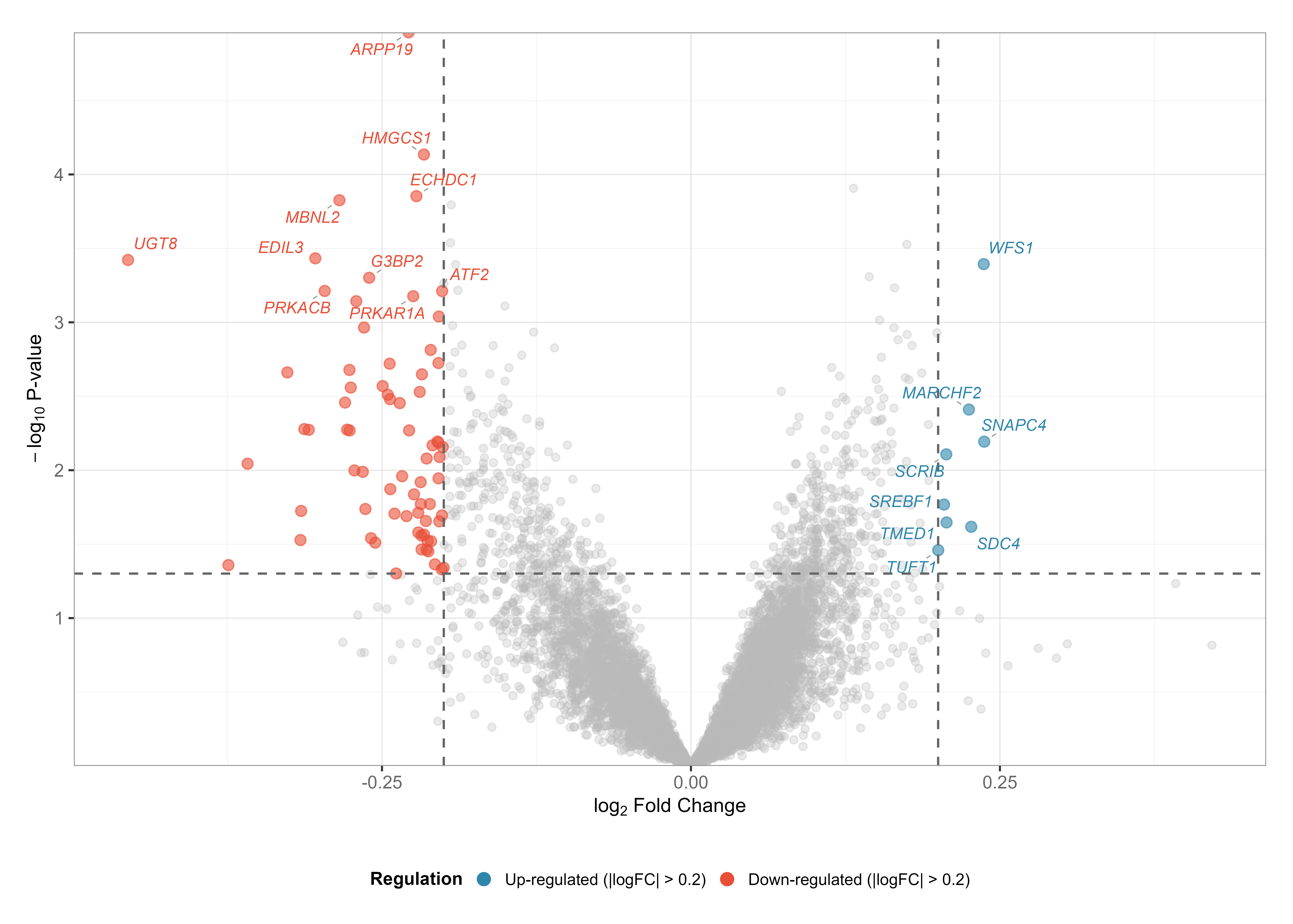

Supplement: Supplementary file 4 — Supplementary Material 4: Volcano plot of the BPD-stratified meta-analysis. Volcano plot showing gene expression changes in the BPD versus control meta-analysis. Blue dots indicate upregulated genes and red dots indicate downregulated genes meeting the nominal thresholds of p < 0.05 and |logFC| > 0.2. Grey dots represent non-significant genes. Dashed lines indicate the significance and fold-change cutoffs. [file 12888_2026_8170_MOESM4_ESM.png]

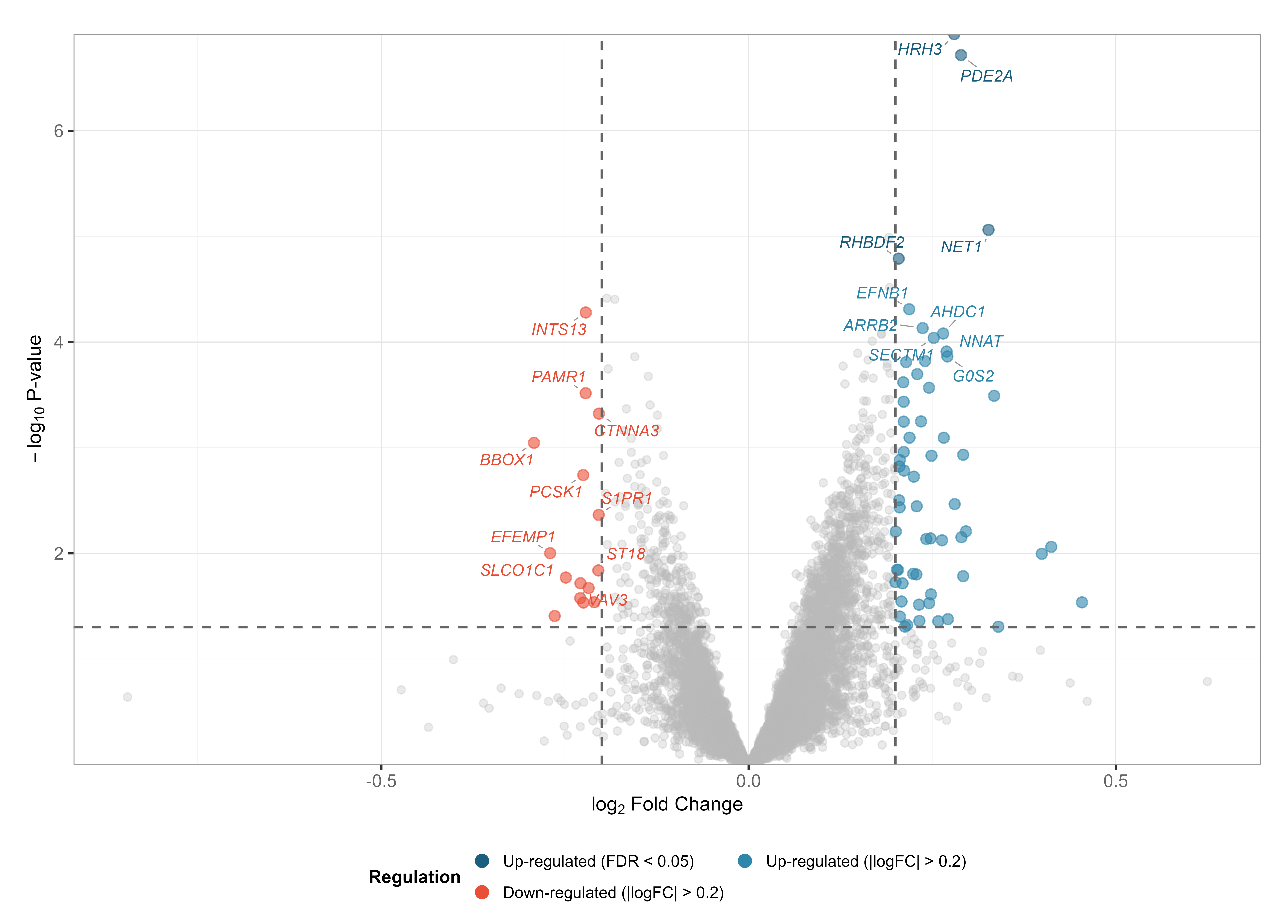

Supplement: Supplementary file 5 — Supplementary Material 5: Volcano plot of the MDD-stratified meta-analysis. Volcano plot showing gene expression changes in the MDD versus control meta-analysis. Dark blue dots indicate genes that remained significant after FDR correction, whereas light blue and red dots indicate nominally significant upregulated and downregulated genes meeting the thresholds of p < 0.05 and |logFC| > 0.2. Grey dots represent non-significant genes. Dashed lines indicate the significance and fold-change cutoffs. [file 12888_2026_8170_MOESM5_ESM.png]

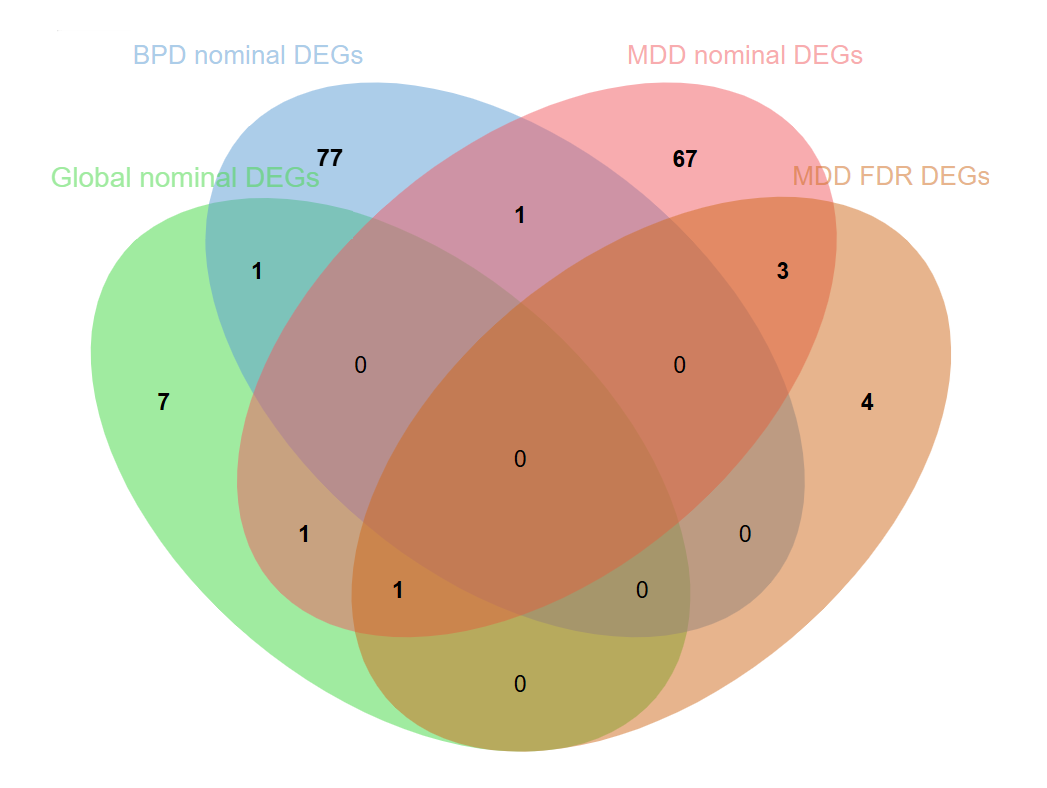

Supplement: Supplementary file 6 — Supplementary Material 6: Overlap of nominal and FDR-significant differentially expressed genes across global and diagnosis-stratified analyses. Venn diagram showing the overlap among nominally differentially expressed genes identified in the global meta-analysis, the BPD-stratified analysis, and the MDD-stratified analysis, together with the FDR-significant genes from the MDD-stratified analysis. [file 12888_2026_8170_MOESM6_ESM.png]

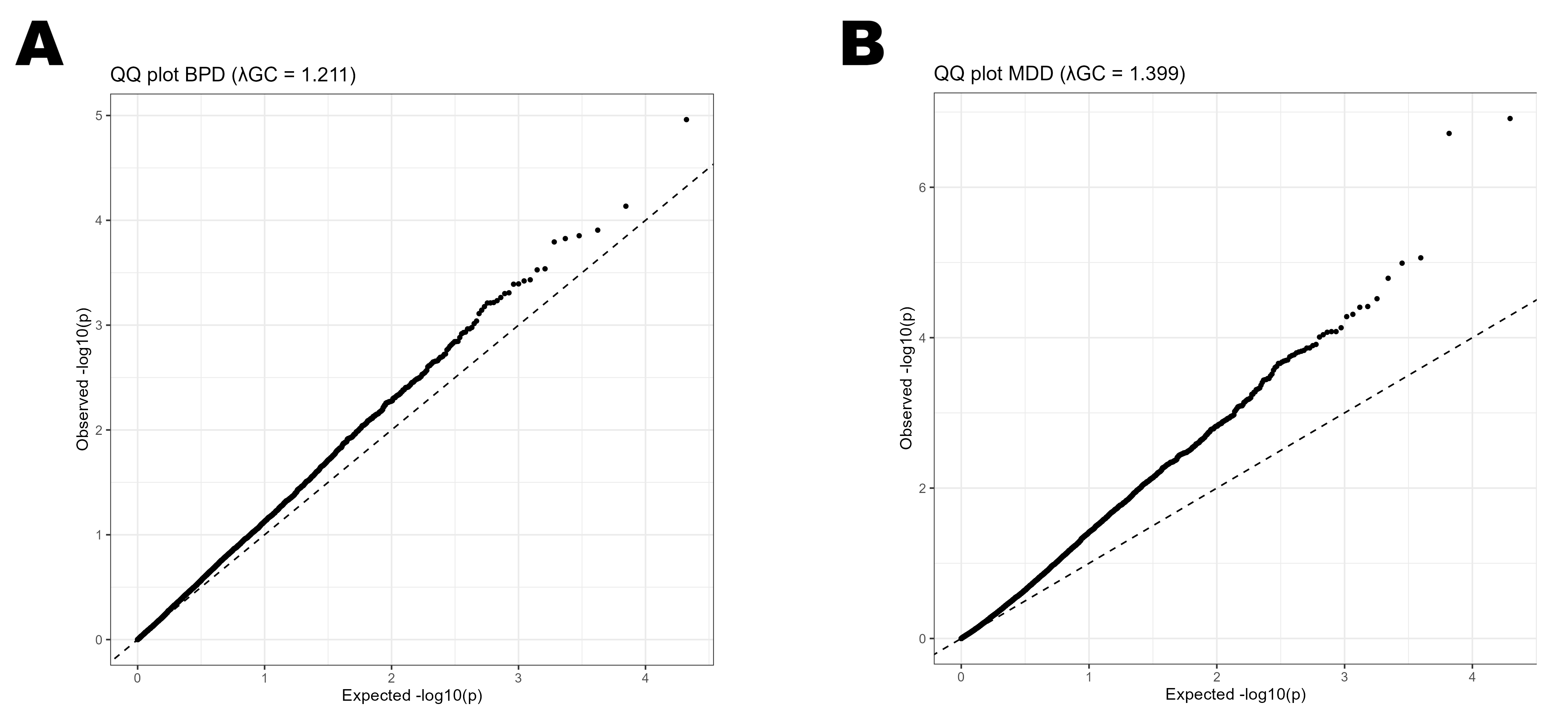

Supplement: Supplementary file 7 — Supplementary Material 7: Quantile–quantile plots of the diagnosis-stratified meta-analyses. QQ plots comparing observed and expected –log10(p) values for (A) the BPD versus control meta-analysis and (B) the MDD versus control meta-analysis. Dashed lines indicate the expected null distribution, and λGC denotes the genomic inflation factor. [file 12888_2026_8170_MOESM7_ESM.png]

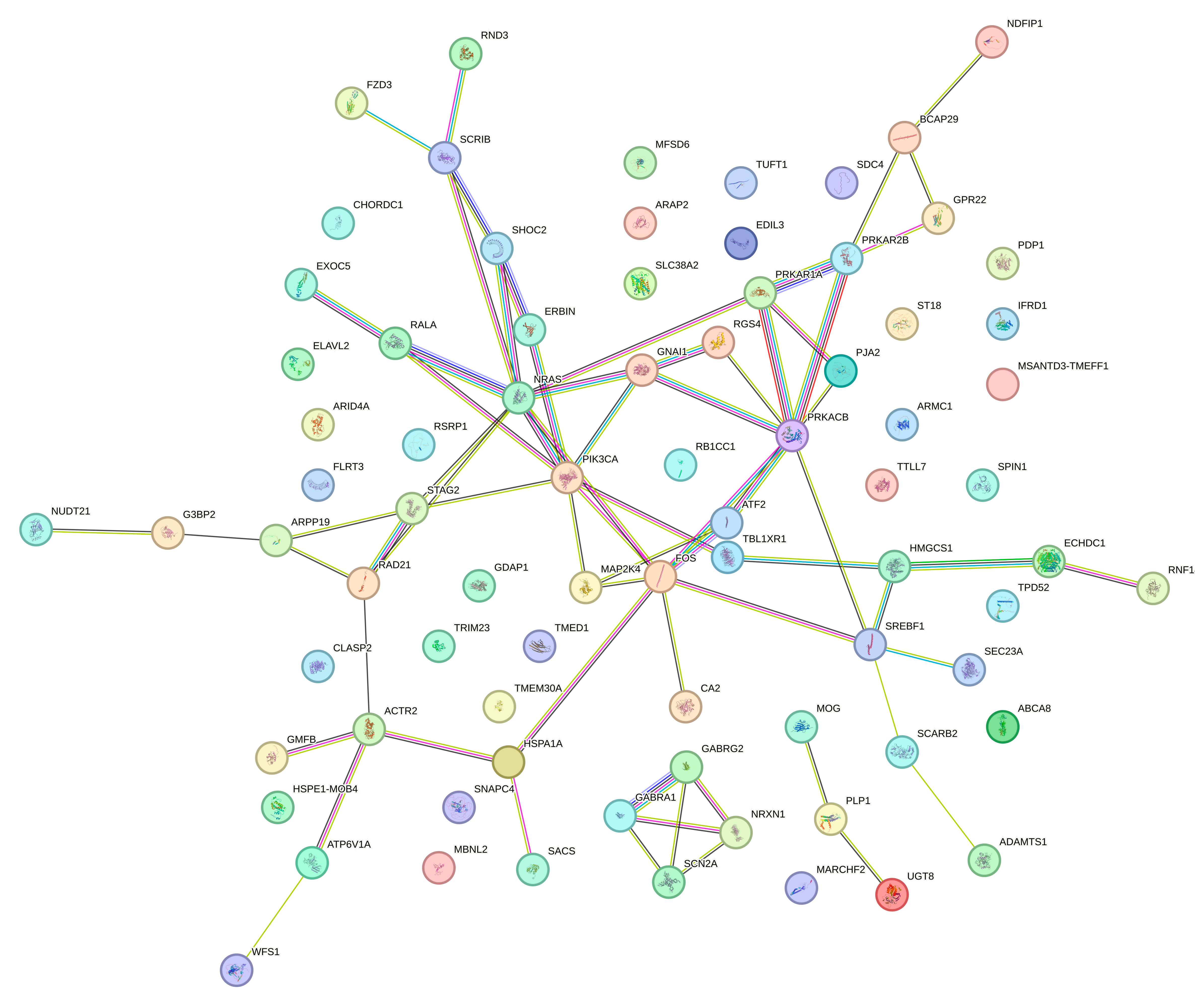

Supplement: Supplementary file 10 — Supplementary Material 10: PPI network of nominally differentially expressed genes in the MDD-stratified meta-analysis. Network generated with STRING using the nominally differentially expressed genes identified in the MDD versus control meta-analysis and a combined interaction score threshold of ≥ 0.4. Nodes represent genes and edges represent predicted or known protein–protein interactions. [file 12888_2026_8170_MOESM10_ESM.png]

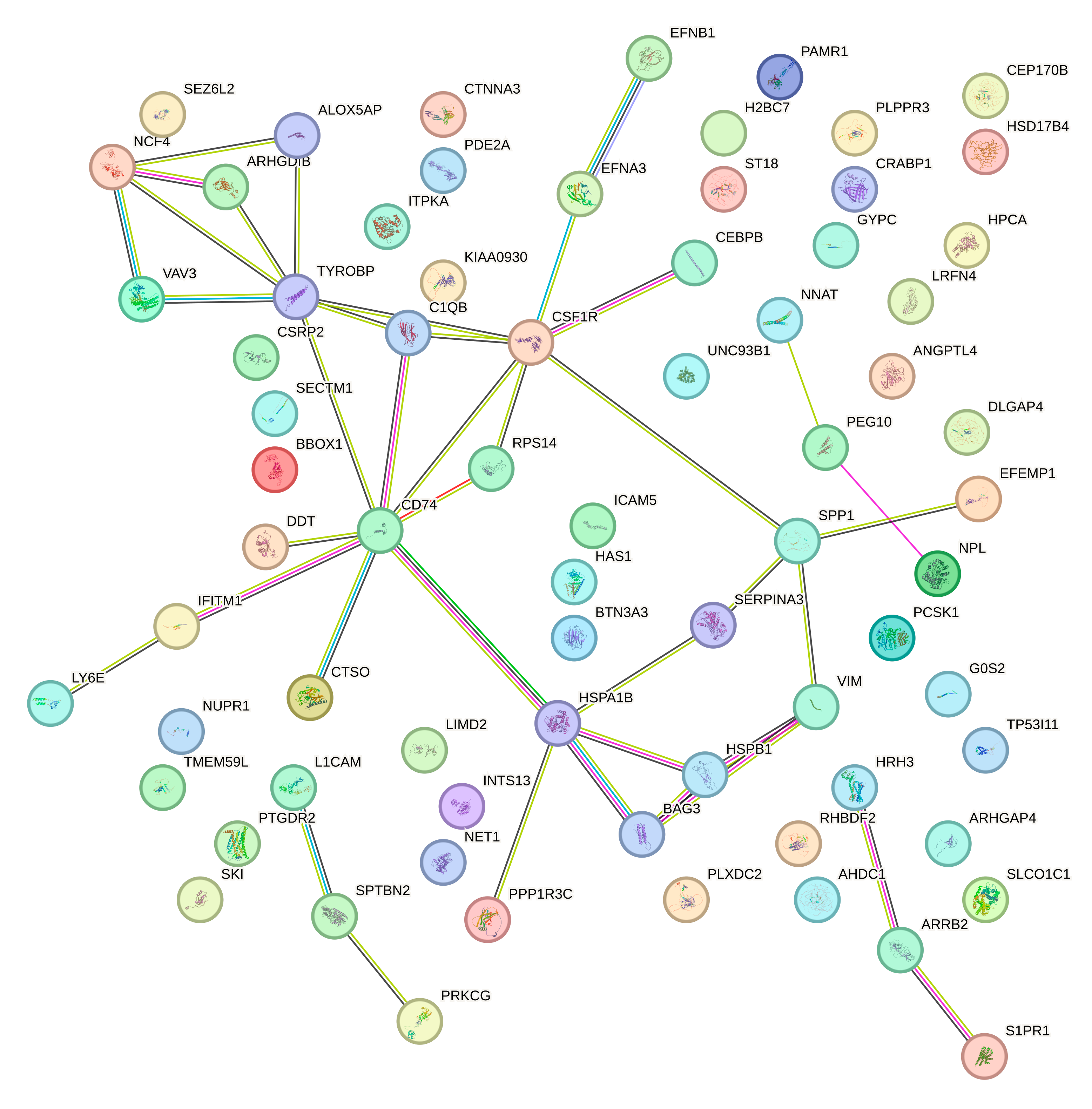

Supplement: Supplementary file 11 — Supplementary Material 11: PPI network of nominally differentially expressed genes in the BPD-stratified meta-analysis. Network generated with STRING using the nominally differentially expressed genes identified in the BPD versus control meta-analysis and a combined interaction score threshold of ≥ 0.4. Nodes represent genes and edges represent predicted or known protein–protein interactions. [file 12888_2026_8170_MOESM11_ESM.png]
